# When reporting *Nocardia* spp is not enough. Brain abscess caused by *Nocardia farcinica*

**DOI:** 10.1099/acmi.0.000091

**Published:** 2020-01-20

**Authors:** J. Zintgraff, M. Prieto, M. Peña, F. Simoiz, S. Rosenblit, D. D'Alessandro, A. Fernandez Garces, V. Di Matteo, R. Astesana, M. Panno

**Affiliations:** ^1^​ Servicio de Bacteriología. Clínica AMEBPBA, CABA, Argentina; ^2^​ Servicio de Bacteriología Clínica. INEI ANLIS “Dr Carlos G. Malbrán”, CABA, Argentina; ^3^​ Servicio de Bacteriología Especial. INEI ANLIS “Dr Carlos G. Malbrán”, CABA, Argentina; ^4^​ Servicio de Clínica Médica - Clínica AMEBPBA, CABA, Argentina; ^5^​ Servicio de Infectología - Clínica AMEBPBA, CABA, Argentina; ^6^​ Coordinador de Laboratorio - Clínica AMEBPBA, CABA, Argentina; ^7^​ Dirección Médica- Clínica AMEBPBA, CABA, Argentina

**Keywords:** Nocardia species, MALDI-TOF, brain abscess

## Abstract

Abscesses caused by the genus *Nocardia* spp are relatively rare, accounting for approximately 2 % of all brain abscesses, but with a significantly higher mortality. Special stains of brain abscess material from a 60-year-old man showed Gram-positive branching bacilli and the presence of long, acid-fast branching filamentous bacilli suggesting *Nocardia* infection. Presented here is a case of multidisciplinary management of a patient who developed cerebral abscesses by *Nocardia farcinica*, confirmed by matrix-assisted laser desorption/ionization time-of-flight mass spectrometry (MALDI-TOF MS), that was susceptible to trimethoprim/sulfamethoxazole, linezolid, imipenem and not susceptible to minocycline. This case highlights the importance of performing subtyping and antimicrobial testing in order to improve clinical and treatment outcomes due to patterns of antibiotics resistance among *Nocardia* species.

## Introduction

Nocardiosis can be a localized or disseminated infection, caused by an aerobic Actinomyces species of *Nocardia*, which is encountered worldwide. Infections caused by *Nocardia farcinica* are potentially lethal because of the organism’s tendency to disseminate throughout a patient and to resist antibiotics [1]. Infection usually occurs through inhalation or direct inoculation by surgery or skin trauma [2]. Most cases of central nervous system (CNS) complications are considered a disseminated infection from a lung focus [3]. *Nocardia* brain abscess is rare and accounts for 1–2 % of all cerebral abscesses [4]. However, it is the most common secondary infection due to *Nocardia* spp and has a high mortality rate, which can be as high as 20 % in immunocompetent patients and 55 % in immunocompromised patients respectively. In cases of multiple abscesses, the mortality rate rises to 66 % [5]. Herein, we report a case of *Nocardia farcinica* brain infection, highlighting the importance of appropriate subtyping and susceptibility testing of these bacteria.

## Case report

**Fig. 1.**
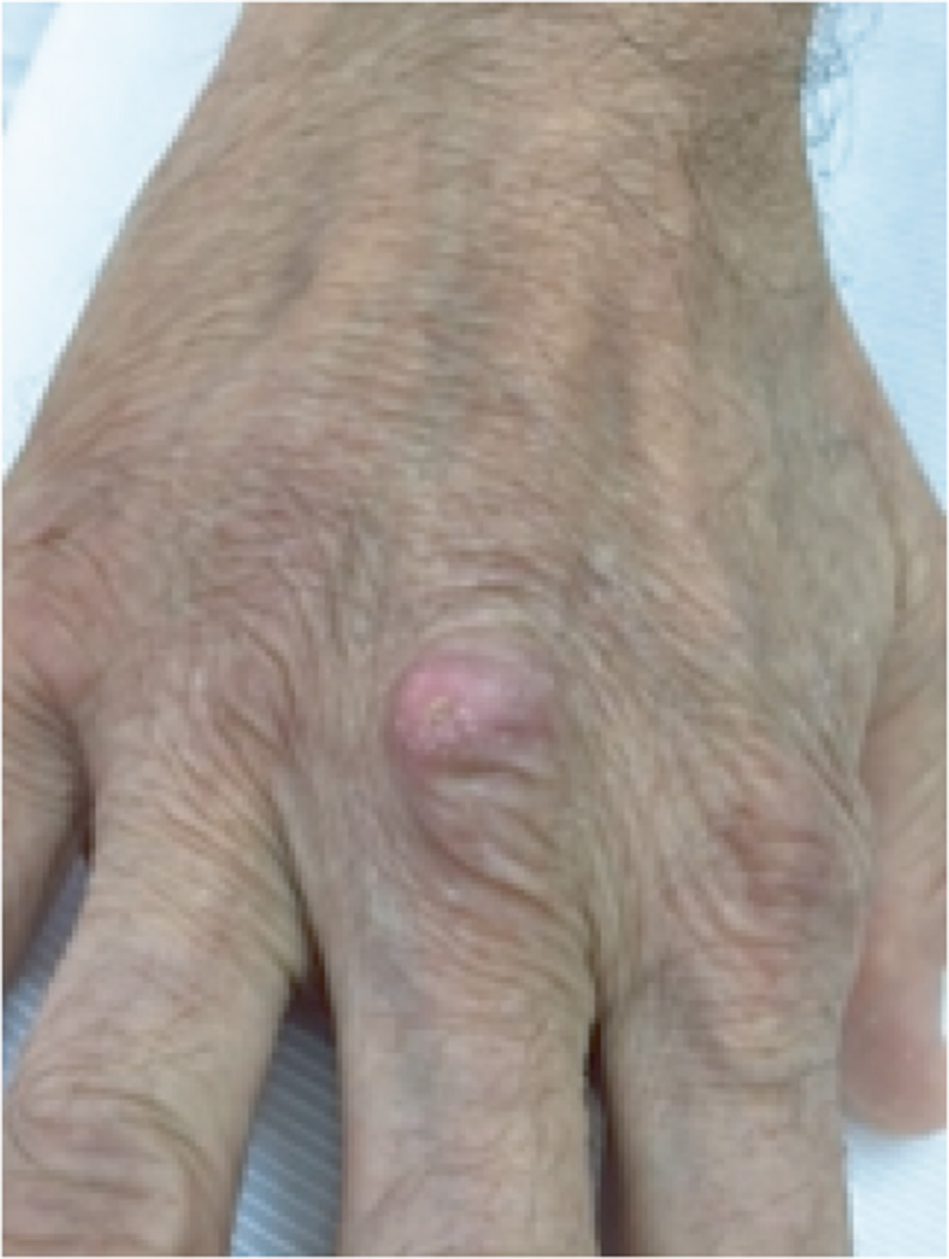
Cutaneous nodule in the right hand without phlogosis.

**Fig. 2.**
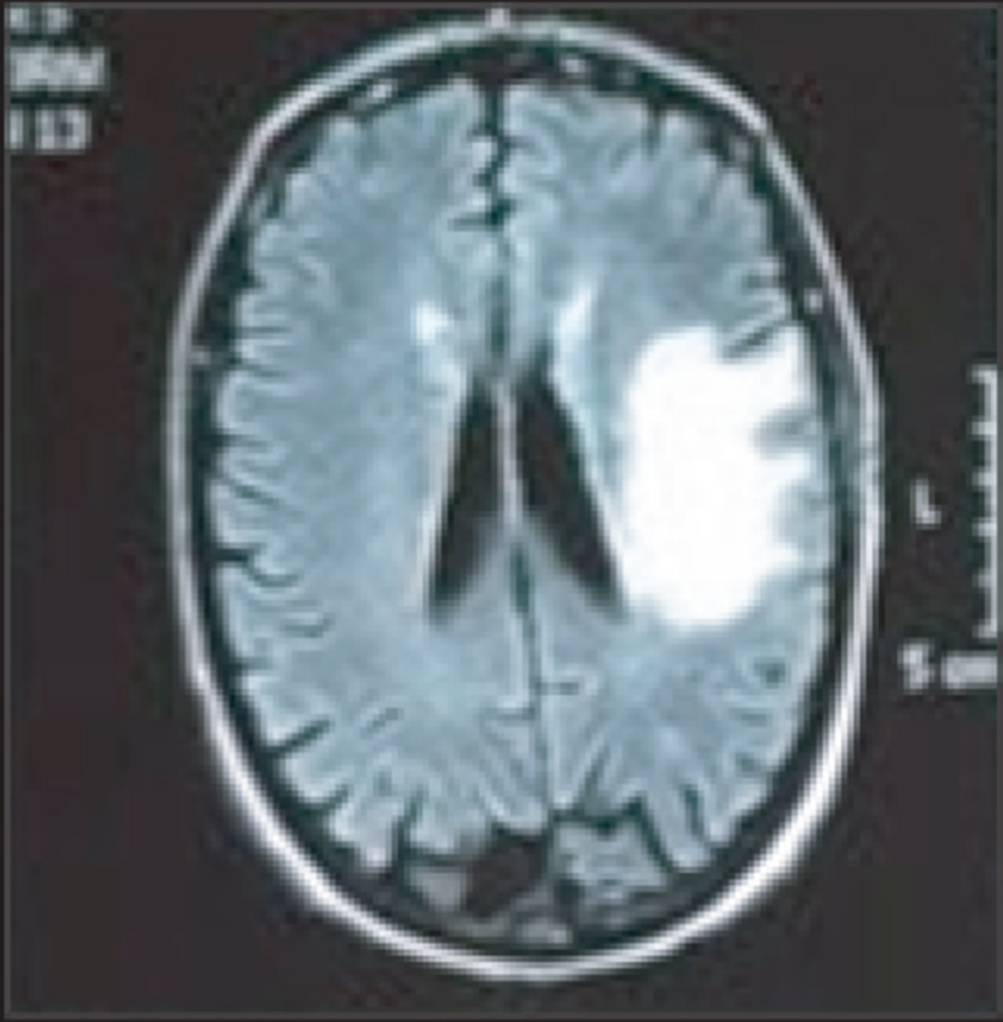
Gadolinium–enhanced magnetic resonance imaging of the brain.

**Fig. 3.**
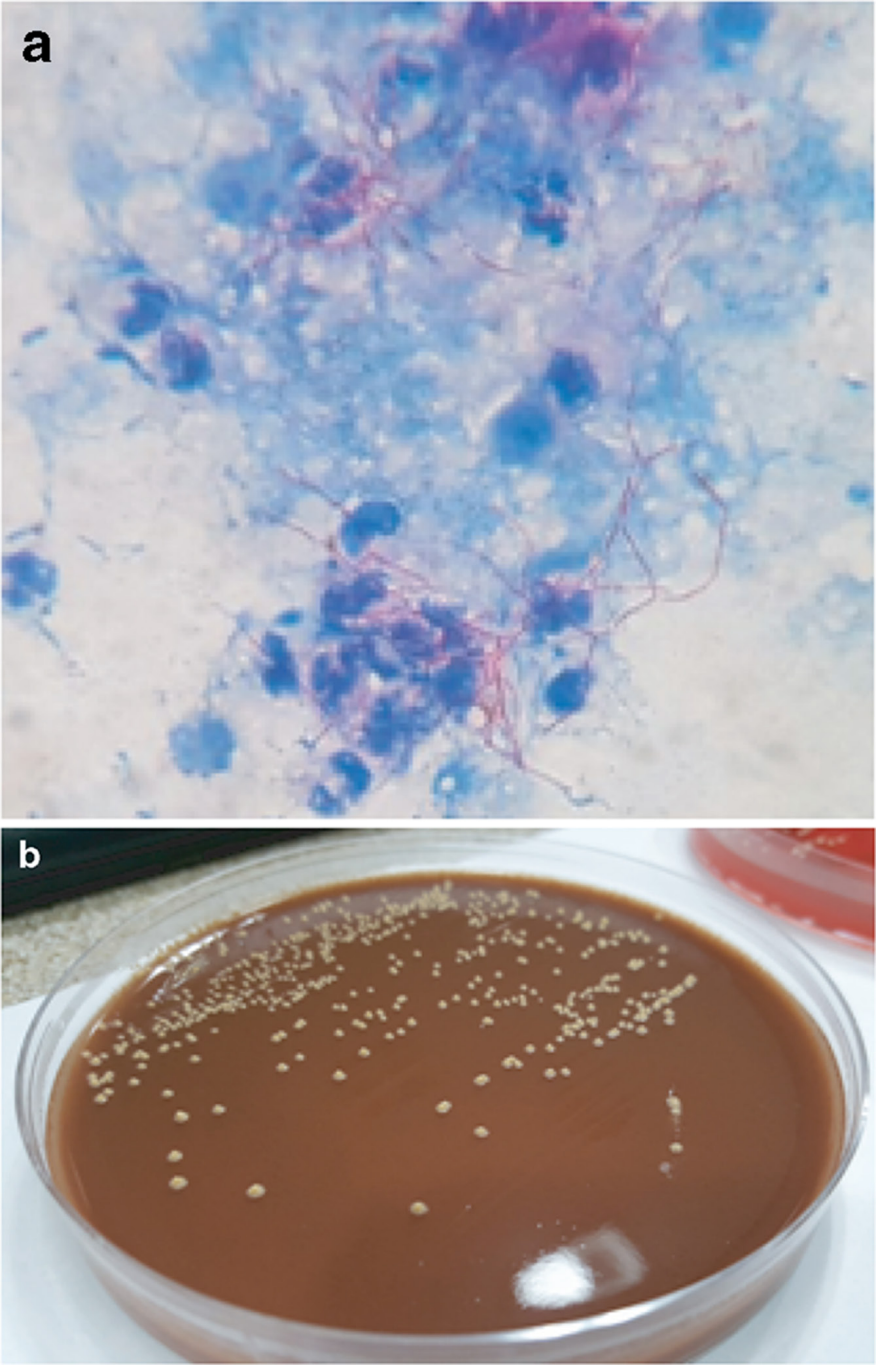
(a) Modified Ziehl-Neelsen (ZN) staining (oil immersion objective, 1000×) of brain abscess showing the presence of long, acid-fast branching filamentous bacilli in a background of many polymorphonuclear leukocytes suggestive of *Nocardia* spp. (b) Rough yellow colonies grown on chocolate blood agar.

A 60-year-old male with a history of autoimmune hepatitis treated with prednisolone (10 mg day^−1^), arrived at our clinic with aphasia and dysarthria which evolved into a fever episode. Upon physical examination, a cutaneous nodule was observed on his right hand without phlogosis (Fig. 1). On admission, laboratory evaluation revealed hemoglobin 13.3 g dl^−1^, hematocrit 38.1%, white blood cell count 11 000 mm^−3^, and platelets 198 000 mm^−3^. Chemistry findings were blood urea nitrogen 33 mg dl^−1^, creatinine 1.11 mg dl^−1^, and glucose 93 mg dl^−1^, C-reactive protein <6 mg dl^−1^. He was negative for human immunodeficiency virus. A computed tomography (CT) scan revealed a pleural base consolidation lesion in the left upper lobe with a small intralesional cavity; thus, empiric treatment for community-acquired pneumonia with ampicillin-sulbactam (1.5 g IV every 6 h) was initiated. Bronchoalveolar lavage cultures for bacteria, mycobacteria, fungi and nocardia were negative. Two days later the patient was still febrile and confused so a gadolinium–enhanced magnetic resonance imaging of the brain (brain MRI) was requested: focal images showed dense perilesional edema on the left parietal, compatible with abscess (Fig. 2), thus an aspiration of the material was performed. Gram and Modified Ziehl-Neelsen stain, done in parallel, of the aspirated material revealed Gram-positive branching bacilli and the presence of long, acid-fast branching filamentous bacilli (Fig. 3a). Three days later, filamentous yellow colonies with aerial mycelia were reported to be growing (Fig. 3b). The isolate was identified as *N. farcinica* by MALDI-TOF (Microflex LT Bruker Daltonics, Bremen, Germany), score 2.009, with no other species in the top ten list report, at the National Reference Laboratory; and identity was confirmed by standard biochemical tests according to the literature [6](Table 1).

**Table 1.** Phenotypic characteristics of selected *Nocardia* species known to cause human disease.^*a*^

Species	Growth at 45 °C	Production of:			Hydrolysisof:						Utilizationof:			
		Arylsulfatase (14 days)	Nitrate reductase	Urease	Adenine	Casein	Esculin	Hypoxanthine	Tyrosine	Xanthine	Acetamide*^b^*	Citrate*^c^*	l-Rhamnose*^c^*	d-Sorbitol*^c^*
*** N. abscessus ***	−	−	+	+	−	−	−	−	−	−	−	+	−	−
*** N. africana ***	+	+	−	−	−	+	−	−	−	−	na	−	−	−
*** N. aobensis ***	−	na	na	+	−	−	na	−	−	−	na	−	−	−
*** N. asiatica ***	−	−	+	V	−	−	−	−	−	−	na	+	+	−
*** N. beijingensis ***	−	na	na	+	−	−	−	−	−	+	na	+	+	+
*** N. brasiliensis ***	−	−	+	+	−	+	+	+	+	−	−	+	−	−
*** N. brevicatena ***	−	−	−	−	−	−	+	−	−	−	−	−	−	−
*** N. carnea ***	−	na	+	−	−	−	+	−	−	−	na	−	−	+
*** N. cyriacigeorgica ***	+	na	+	−	−	−	−	−	−	−	+	−	−	−
***N. farinica***	+	−	−	+	−	−	+	−	−	−	+	−	+	−
*** N. kruczakiae ***	+^*d*^	+	na	na	−	−	+	−	−	−	−	+	−	−
*** N. mexicana ***	−	−	na	+	+	−	na	+	−/W	−	na	na	+	+
*** N. niigatensis ***	−	na	na	V	−	−	na	w	−	−	na	−	−	−
*** N. nova ***	−	+	+	+	−	−	−	−	−	−	−	−	−	−
*** N. otitidiscaviarum ***	V	na	+	+	−	−	+	+	−	+	−	−	−	−
*** N. paucivorans ***	+	na	−	+	−	−	+	−	−	−	−	−	−	−
*** N. pseudobrasiliensis ***	−	−	−	+	+	+	+	+	+	−	na	+	−	−
N.***N. transvalensis complex***	V	−	+	+	−	−	+	+	−	−	−	+	−	V
*** N. veterana ***	+	−	−	+	−	−	−	−	−	−	−	−	−	−

*a,* Symbols and abbreviations: −, negative; +, positive; na, not available; V, variable; w, weak.

*b*, Utilization as sole source of carbon and nitrogen.

*c*, Utilization as sole source of carbon.

*d,* Optimal growth.

Antibiotic susceptibility testing showed the sensitivity of the cultured bacterium to trimethoprim/sulfamethoxazole (TMP/SMX) (minimum inhibitory concentration [MIC]=0.125 mcg ml^–1^), linezolid (MIC=1 mcg m^–1^), imipenem (MIC=1 mcg m^–1^), and not susceptible to minocycline (MIC=2 mcg ml^–1^), according to the Clinical and Laboratory Standards Institute guidelines : *Susceptibility Testing of Mycobacteria, Nocardiae, and other Aerobic Actinomycetes;* M24-A2, 2011 [7] and the characteristic *N. farcinica* pattern of resistance to tobramycin, ceftriaxone and clarithromycin [8]. Empiric treatment was stopped and he was started on TMP/SMX (160/800 mg qid) and Linezolid (600 mg/bid). After 2 weeks of treatment, the size of the abscess decreased significantly and he was discharged from the institution with TMP/SMX (160/800 mg qid) as oral therapy.

## DISCUSSION

An increasing number of *N.farcinica* infections have been reported due to recent improvements in taxonomy and diagnostic methodologies. This has implications for therapy because of the organism's pathogenicity and natural resistance to third-generation cephalosporins (usually used as empiric treatment for brain abscess). Even though the amplification and sequencing of the bacterial rRNA (16S rRNA or *hsp65* gene) [9–11] allows for the rapid identification of the *Nocardia* species, these molecular studies are unfortunately often limited to research laboratories. Two methodologies were used is this case: biochemical tests and MALDI-TOF (matrix-assisted laser desorption/ionization time-of-flight) mass spectrometry. Regarding the latter, a recent publication [12] indicates that for some *Nocardia* species identification, results obtained through MALDI-TOF are becoming more reliable and faster, making the technology a rapid and accurate identification tool which can replace sequencing in a clinical microbiology laboratory.

In conclusion, the course of *Nocardia* brain abscesses are unpredictable and the patient's condition may deteriorate quickly. It is important to emphasize that no therapeutic guidelines are available because of the different antimicrobial sensitivity patterns among *Nocardia* species; that is why both appropriate subtyping and susceptibility testing of uncommon species could expedite the diagnosis and facilitate treatment to reduce morbidity and mortality.
